# Case of Extirpation of the Thyroid Gland

**Published:** 1835

**Authors:** 


V. *Case of Extirpation of the Thyroid Gland.* By N. R. Smith, M. D., Professor of Surgery in the University of Maryland. (N. A. Archives, for Aug. 1835.)

Professor Smith tells us that "the origin of the disease was as far back as 1815, beginning *at no time*." What was its duration? Patient about 40 years of age, the mother of several children, sickly aspect—had lived many years "in a malarious" district, and had a *severe bilious fever one year before the operation*. The tumified gland extended from the os hyoides downwards, so as to hang over the margin of the sternum. A drawing which accompanies the case is said to afford a correct "idea of its size and form"—to our apprehension the tumor is represented as lying with its lower portion two or three inches on the sternum.

"The general configuration of the tumor, in short, was that of common goitre, and *when* the integuments were entire, it presented the varicose veins usually conspicuous in that disease."

We suppose the above sentence is intended to convey the opinion, that this was a case of goitre. But Professor S. did not see the varicose veins, he saw the gland greatly enlarged and ulcerated; and it could not be healed. There was now issuing from the breast a serous fetid discharge, and a fungus having "*a strong resemblance to fungus hæmatodes.*"

Professor Geddings joined in the opinion that this was an enlarged thyroid gland, the right lobe being chiefly concerned. The superior arteries could, as is usual, be felt passing down to the gland. The gentlemen satisfied themselves that "the great vessels of the neck were not involved in, or adherent to the tumor." (It was found afterwards that they were adherent to the tumor.) That the attachments were slight, was, we suppose, to be expected, owing to the tumor's having long been dragging down towards the sternum.

It was determined, in consultation, that the only resource that afforded any prospect, was the entire removal of the disease by extirpation. But, we might ask, who has the tact for removing *entirely an ulcerated malignant tumor*, which this certainly was?

Some time after 1815, or on — of ——, Professor Smith performed the operation. "An elliptical incision, the length of which corresponded with the sack," was made. The dissection was now commenced on the right side, and so directed as to aim at tying the superior thyroid artery on that side: it became necessary to tie it more than once, its tortuous course bringing it under the knife.

In turning back the sterno-mastoid muscle, which lay hard upon the tumor, the anterior jugular vein was cut. The vein being emptied of its blood, by unavoidable pressure, the operator became apprehensive of great risk, from the entrance of air into the wounded vein, and strove to avoid the accident, by compressing the vessel while tying it. The utmost care did not serve to prevent the admission of a small portion of air into the vein, and excited great anxiety in the mind of Prof. S., but no visible evil arose from it, though three portions were admitted during the operation.

It will be recollected that a fatal accident of this kind befel Dupuytren, in operating upon a tumor of the neck. This case is sufficient to convince us that such accidents may occur; they are, however, fortunately very rare; and Prof. S. having seen this occurrence three times previously, and three times in the present operation is strikingly singular. We have seen large veins again and again laid open, in attempts at suicide—in one instance the external jugular was divided, and the patient had nearly bled to death, no air was drawn in or suffered to enter. We have often removed large tumors of the neck, but have not seen any thing of the occurrence in view. Our memory does not furnish us with more than one fatal accident of this kind, and how many hundreds of tumors have been removed? how many wounds inflicted upon the neck? Besides, Professor Smith's own experience, on this point, shows that danger can only arise from carelessness, since he witnessed the entrance of air on four different occasions, and no evil followed—why then his great fears from the ingress of air into veins? We would not, however, wish to divert attention from this source of danger, be it ever so slight; nor to excite fearful apprehensions which may tend to embarrass operators, when it is sufficient that they keep the risk in view, and calmly avert the possible danger, by compression on the cut ends, but by no means tying all the veins.

We are reminded here of another risk from wounds of the veins of the neck. We were once called to a man, who, in a desperate attempt to commit suicide, drew a razor, with a rounded cutting end, across the throat, so as to make a considerable wound, and then thrust the end of the razor down into the left side of the neck, till it reached the cervical vessels, and, turning it round, he uncovered the carotid artery, a little below its bifurcation. Great hemorrhage took place, and he lost several quarts of blood, it was supposed, before we arrived. The parts being much deranged, and injected with blood, we found it necessary deliberately to dissect, so as to lay bare the great cervical vessels, and now we found that they were not cut, and that all this vast and dangerous hemorrhage came from the external jugular vein. We applied an animal ligature, and the hemorrhage was arrested at once. We saw this case in consultation with our friend, Dr. S. B. Martin. Now although hemorrhage may be said to be slow from veins, compared to that of arteries; yet, it is sometimes such as to lead to danger, and should not be lost sight of, particularly when the surgeon cannot remain near his patient.

The dissection having been carried through the more superficial vessels, it now became more easy, particularly on the left side. On this side was heard the ingress of air again, and induced the operator to hold each portion of structures between the thumb and fingers, while they were divided—the lower portions of veins were secured by ligatures. Can it be necessary, after so many successful operations, upon the neck, to tie all the veins? Is there not considerable risk of phlebetis? I should have no hesitation in trusting to a careful pressure of the ends of cut veins, for a minute or two, between the thumb and forefinger, unless there be some thickening of the venous coats, which may cause the vessel to remain open; and, I strongly suspect, that some degree of density of the coats must exist, to give rise to a dangerous gurgation of air. If we do tie, the animal ligature should be used.

"The sheath of the great vessels on each side lay in immediate contact with the tumor, and, indeed, was to some extent incorporated with it. I also found that the fascia covering the thyroid and cricoid cartilages, was thickened, and adherent to the tumor. Its separation from these connections was the most difficult and tedious part of the operation. The separation of the tumor from the parts immediately above the border of the sternum, where I was prepared to expect most difficulty and danger, was effected with infinitely more ease than I had anticipated." The narrative goes on to state, that the vessels were elongated, contracted in their volume, and pulsating feebly; many veins obliterated; the right inferior thyroid artery cord-like, the left missing. A few branches of vessels were secured, in the lower part of the wound; the trachea was nearly laid bare down to the sternum.

It should be remembered that any anomaly in the large arteries, at this point, might be a source of danger—the high bifurcation, which we sometimes see in the arteria-innominata, might create considerable difficulty and risk.

Looking upon the wound, the great vessels of the neck were seen beating—the recurrent laryngeal nerve was seen on the right—the trachea bare. Where was the œsophagus? The wound was closed with the interrupted suture, and dressed, leaving the patient pretty comfortable during the first day. The next morning considerable hemorrhage took place; the wound was opened, and a ligature applied to a small artery, lint and alum to less important vessels. The hemorrhage seems to have prostrated the patient considerably, and, together with the distress of re-dressing, produced great prostration. "She did not swallow without effort, but without difficulty."

The patient was supposed to be doing well, until the fifth day, when a severe rigor, followed by fever, took place. *The breathing was sonorous and embarrassed*, cough and irritability of stomach existed. Are these principal symptoms usual concomitants of bilious fever? But the paroxysm passed off, with "a sweating stage, *precisely as a paroxysm of fever*. The wound, which had suppurated kindly, now began to exhibit a flabby appearance, and to discharge an unhealthy secretion."

"The chills were evidently of malarious origin, and the patient undoubtedly came to me predisposed to intermittent fever. To this, in part, I think I may with propriety ascribe the fatal result."

It would perhaps be thought invidious to deprive Professor Smith of the pleasure of getting out at this loop-hole. But we must be allowed to express our doubts, whether a patient greatly prostrated, by ulcerated fungus tumor, of long duration, was not more likely to die of the effects of the operation, than from intermittent fever, simply because she had lived in "a malarious district," although she had had no disease ascribable to malaria for a whole year prior to the operation. Why is not the period of the year made to fill up two blanks, in the report of the case? If such an operation would not make a patient undergo sweating stages and fever, and even death, one cannot well conceive what could so affect a human body.

We are at all times willing to let practitioners make their own decisions, and, occasionally, boldly step forward to hazard a difficult and dangerous operation, where there is any reasonable hope of saving a patient, but let them always call things by their right names. We, looking on the result, say, that in such a habit, with a disease so protracted, and so evidently malignant, and beyond the proper period for operation, the patient ought not to have been subjected to so formidable an operation: apart from this important consideration, Prof. Smith has performed a difficult, and, speaking of the execution merely, splendid operation; but, we think, if he had left the patient to the intermittent fever single-handed, it would have been better for her, and just as well for surgery. We admonish our younger friends, in the profession, not to grapple with ulcerated malignant tumors of the thyroid glands, otherwise they may lose all such patients, even without the consolation of *hoping* they may have died, "in part," of intermittent fever.

Professor Smith would offer this case in support of the opinion that the dangers of extirpating the thyroid gland have been overrated. But does not the narrative of the case show that the vessels of greatest difficulty, nay, the only ones of difficulty, were not in their normal condition; on the contrary the right thyroid artery pulsated feebly, was hard and elongated; the left, of all things the most difficult to manage in the removal of the thyroid gland, was wanting—shall we, nevertheless, admit this as a good proof of the safety, and unexpected facilities attending the performance of the operation? What does this operation prove? That an enlarged fungating thyroid gland may be removed; all the vessels laid bare as well as the nerves; vein after vein tied; the trachea denuded throughout its whole length; the œsophagus exposed; and, still the patient may bear the operation; but it does not prove that recovery may or can take place. If we admit that it was meritorious to hazard much to rescue a deserving woman from death, the result shows that under similar circumstances it should never be repeated.

Our author seems desirous of having it understood that he extirpated the entire gland. If he has given an accurate description of the operation, which we admit, there can be no doubt on this point; but did he take away all the contaminated parts? The termination shows what we may know, *a priori*, that it was not, nay, could not be done.

As to the dangers of the extirpation of this gland, we know that among the earlier successful attempts, may be mentioned those of Desault, who tied the thyroid arteries, as the first step of the operation; and Mr. Gooch, who saved his patient by making compression, by the aid of a succession of fingers, for eight days.

What shall we say to the declaration that "the above case appears to me to warrant the belief, that it may be undertaken with hope of success"? Now, we know that this operation has often been successfully performed, under proper circumstances. Does it follow, that it will succeed when performed under improper circumstances? Read the history of ulcerated tumors, "resembling fungus hæmatodes," and look at the result of the case under review, and then tell us, who is the man to repeat this operation, under like circumstances; or say, that, inasmuch as intermittent fever may have come to aid the business of death in this case, therefore, it may be held up as worthy of a place among our precedents in surgery. Our calmer reflections will not allow it such a place. H. G. J.

